# Cytotoxic and Pro-Apoptotic Effects of a Sub-Toxic Concentration of Fluvastatin on OVCAR3 Ovarian Cancer Cells After its Optimized Formulation to Melittin Nano-Conjugates

**DOI:** 10.3389/fphar.2020.642171

**Published:** 2021-02-03

**Authors:** Shaimaa M. Badr-Eldin, Nabil A. Alhakamy, Usama A. Fahmy, Osama A. A. Ahmed, Hani Z. Asfour, Abdulhamid A. Althagafi, Hibah M. Aldawsari, Waleed Y. Rizg, Wael A. Mahdi, Adel F. Alghaith, Sultan Alshehri, Filippo Caraci, Giuseppe Caruso

**Affiliations:** ^1^ Department of Pharmaceutics, King Abdulaziz University, Jeddah, Saudi Arabia; ^2^ Department of Pharmaceutics and Industrial Pharmacy, Cairo University, Cairo, Egypt; ^3^ Advanced Drug Delivery Research Group, King Abdulaziz University, Jeddah, Saudi Arabia; ^4^ Center of Excellence for Drug Research and Pharmaceutical Industries, King Abdulaziz University, Jeddah, Saudi Arabia; ^5^ Mohamed Saeed Tamer Chair for Pharmaceutical Industries, King Abdulaziz University, Jeddah, Saudi Arabia; ^6^ Department of Medical Microbiology and Parasitology, King Abdulaziz University, Jeddah, Saudi Arabia; ^7^ Department of Clinical Pharmacy, King Abdulaziz University, Jeddah, Saudi Arabia; ^8^ Department of Pharmaceutics, College of Pharmacy, King Saud University, Riyadh, Saudi Arabia; ^9^ Department of Pharmaceutical Sciences, College of Pharmacy, Almaarefa University, Riyadh, Saudi Arabia; ^10^ Oasi Research Institute—IRCCS, Troina, Italy; ^11^ Department of Drug and Health Sciences, University of Catania, Catania, Italy

**Keywords:** fluvastatin, melittin, nano-conjugates, apoptosis, cytotoxicity, ovarian cancer

## Abstract

Fluvastatin (FLV) is a hydroxymethylglutaryl coenzyme A (HMG-CoA) reductase inhibitor often used to lower total and low-density lipoprotein (LDL) cholesterol and for the prevention of adverse cardiovascular events. This drug as well as melittin (MEL), the major component of honeybee venom (*Apis mellifera*), has shown antineoplastic activity, then representing promising approaches for cancer therapy. However, adverse effects related to the use of FLV and MEL have been reported and very few studies have been carried out to obtain an optimized formulation allowing for combining the two drugs and then maximizing the anticancer activity, then minimizing the needed dosage. In the present study, an optimized formulation in terms of minimized particle size and maximized zeta potential was investigated for its cytotoxic potential in human OVCAR3 ovarian cancer cells. FLV-MEL nano-conjugates, containing a sub-toxic concentration of drug, demonstrated an improved cytotoxic potential (IC50 = 2.5 µM), about 18-fold lower, compared to the free drug (IC50 = 45.7 µM). Cell cycle analysis studies demonstrated the significant inhibition of the OVCAR3 cells proliferation exerted by FLV-MEL nano-conjugates compared to all the other treatments, with a higher percentage of cells accumulating on G2/M and pre-G1 phases, paralleled by lower percentage of cells in G0/G1 and S phases. The synergistic antineoplastic activity of FLV and MEL combined in the optimized formula was also showed by the marked pronecrotic and pro-apoptotic activities, the latter mediated by the modulation of BAX/BCL-2 ratio in favor of BAX. Our optimized FLV-MEL formulation might therefore represents a novel path for the development of specific and more effective antineoplastic drugs directed against ovarian cancer.

## Introduction

Cancer represents the second leading cause of death globally, accounting for almost 10 million deaths, and involves different molecular mechanisms leading to deregulated proliferation of cells (Sung et al., 2012). These uncontrolled cells do not respond appropriately to the signals regulating the physiological cell behavior; instead they grow and multiply “irrationally” and, in the worst scenario, spread to a different body part from where the tumor started forming metastases (Cooper and Hausman, 2000). Numerous intrinsic and non-intrinsic cancer risk factors have been identified including spontaneous DNA mutations, smoking, nutrient intake, and hormone levels (Wu and Zhu, 2018).

According to the information available on Continuous Update Project (CUP) (https://www.wcrf.org/) and Global Cancer Observatory (GCO) (https://gco.iarc.fr/), ovarian cancer currently represents the 8th most common cause of cancer in women and the 18th most commonly occurring cancer overall. Despite the huge amount of research in the field, late diagnosis is still one of the main issues connected with this cancer type, especially because the symptoms are very hard to spot, in part explaining why only 35% of women will live for 10 or more years after diagnosis (Dong et al., 2014). Furthermore, among all the gynecologic tumors, this type of cancer is considered to be the deadliest (Chobanian and Dietrich, 2008; Chandra et al., 2019). Treatment modalities of ovarian cancer often include debulking surgery and chemotherapy, while radiotherapy is rarely used (Chandra et al., 2019). Unfortunately, unwanted side effects due to the high toxicity and/or the length of the therapy have been related to the abovementioned treatments. Based on the above, we investigated a new possible strategy to treat ovarian cancer through the formulation of a molecule with known pharmacological application in other diseases (the so-called drug repurposing), therefore shortening the required preclinical toxicological studies necessary before their approval (Pushpakom et al., 2019).

Since their approval by Food and Drug Administration (FDA), statins, such as fluvastatin (FLV), have been mainly used to treat and/or prevent cardiovascular diseases (CVD) and to maintain low-density lipoprotein (LDL) cholesterol in the blood at physiological levels (Zhou and Liao, 2009; Islam et al., 2017). In the case of CVD, such as coronary heart disease and heart attack, these drugs are not able to cure these pathological conditions; instead they can help prevent them getting worse or recurring in people who have been identified as subjects with a high risk of developing them. Preclinical *in vitro* and *in vivo* studies have also suggested the use of statins for the treatment of different types of ovarian cancer (Couttenier et al., 2017; Akinwunmi et al., 2019). FLV is one of the hydroxymethylglutaryl coenzyme A (HMG-CoA) reductase inhibitors mainly used for the treatments of dyslipidemia and coronary artery disease (Aoki et al., 2012). In addition to these activities, FLV has shown antineoplastic, antimetastatic, and toxic effects in different experimental models for cancer (Zhang et al., 2010; Salis et al., 2016; Fahmy, 2018).

Melittin (MEL), a small water-soluble cationic peptide composed by 26 amino acids, is the major component of honeybee venom (*Apis mellifera*) (Soman et al., 2009). Although many publications have reported the anticancer activity of this peptide both *in vitro* and *in vivo*, its clinical use is disputed due to its non-specific cytotoxicity and hemolytic activity, especially when used at high doses (Shaw and Kumar, 2019). Since MEL is also able to exert other relevant biological activities such as antibacterial, antiviral, and anti-inflammatory activity (Lyu et al., 2019), the development of optimized formulations in which MEL, at very low concentration, is combined with other antineoplastic drugs to obtain a synergistic anticancer activity can be of the utmost importance.

The aim of the present work was to investigate the cytotoxic and pro-apoptotic efficacy of a sub-toxic concentration of FLV on human OVCAR3 ovarian cancer cells after its optimized formulation to MEL nano-conjugates. This specific cell line was selected because it represents an appropriate model system in which to study drug resistance (Sakhare et al., 2014; Afonso de Lima et al., 2020) as well as the toxic potential of statins alone or in combination with other antineoplastic drugs against ovarian cancer (Scoles et al., 2010; Casella et al., 2014; Abdullah et al., 2019; Yarmolinsky et al., 2020). A two-level, three-factor (2^3^) full factorial design was employed for the preparation and optimization of FLV-MEL nano-conjugates. The optimized FLV-MEL formula, containing a sub-toxic concentration of FLV, was then examined in OVCAR3 cells for the determination of half-maximal inhibitory concentration (IC50) values, cell cycle, apoptosis and necrosis analysis, BAX and BCL-2 proteins, and mitochondrial membrane potential (MMP) determination.

## Material and Methods

### Materials and Reagents

FLV was a kind gift of The Egyptian International Pharmaceutical Industries Company (EIPICO, Cairo, Egypt). When not otherwise specified, the chemicals, all of analytical grade, were supplied by Thermo Fisher Scientific Inc. (Pittsburgh, PA, United States) or Sigma-Aldrich Corporate (St. Louis, MO, United States).

### Experimental Design for Preparation and Optimization of FLV-MEL Nanoconjugates

A two-level, three-factor (2^3^) full factorial design was employed for the preparation of FLV-MEL nano-conjugates. FLV concentration (mM, X_1_), MEL concentration (mM, X_2_), and pH (X_3_) were studied as independent variables, whereas particle size (PS, nm, Y_1_) and zeta potential (ZP, mV, Y_2_) were selected as responses (dependent variables) (Table 1).

**TABLE 1:** Independent variables and responses used in 2^3^ full factorial experimental design for the formulation and optimization of FLV-MEL nano-conjugates.

Independent variables	Levels	
	(−1)	(+1)
X_1_: FLV concentration (mM)	1.00	2.00
X_2_: MEL concentration (mM)	1.00	5.00
X_3_: pH	5.50	9.00
Responses	Desirability constraints	
** **Y_1_: particle size (nm)	** **Minimize	
** **Y_2_: zeta potential (mV)	** **Maximize	

Abbreviations: FLV, fluvastatin; MEL, melittin.

The combination of the levels of the independent variables yielded a total of eight formulations as depicted in Table 2. Analysis of variance (ANOVA) test was employed to assess the effects and interactions of the variables on the responses at 95% level of significance using Design-Expert® Software Version 12 (Stat-Ease Inc., Minneapolis, Minnesota, United States). The equations describing the selected factorial model and process order for each response were generated in terms of coded factors.

**TABLE 2:** Experimental runs and the observed responses of FLV-MEL nano-conjugates prepared according to 2^3^ factorial design.

Experimental run #	Independent variables			PS ± SD	ZP ± SD
	FLV concentration (mM)	MEL concentration (mM)	pH		
F-1	2.00	5.00	5.50	138.11 ± 2.23	11.10 ± 0.54
F-2	2.00	1.00	5.50	180.21 ± 2.72	0.05 ± 0.01
F-3	2.00	5.00	9.00	604.36 ± 4.89	6.50 ± 0.12
F-4	1.00	1.00	9.00	544.31 ± 3.99	0.87 ± 0.04
F-5	1.00	5.00	9.00	597.78 ± 3.12	12.76 ± 0.31
F-6	2.00	1.00	9.00	638.19 ± 4.27	0.05 ± 0.02
F-7	1.00	1.00	5.50	127.25 ± 1.89	0.20 ± 0.03
F-8	1.00	5.00	5.50	160.70 ± 1.69	17.41 ± 0.42

Abbreviations: FLV, fluvastatin; MEL, melittin; PS, particle size; ZP, zeta potential.

The desirability function that consolidates all the investigated responses to anticipate the optimum levels of the independent variables was calculated to select the optimal formulation. The desired goals were set at minimizing the particle size and maximizing the magnitude of the zeta potential (Table 1).

### Determination of IC50 by MTT Assay

OVCAR3 ovarian cancer cells were cultured as described elsewhere (Alhakamy et al., 2020). The IC50 values of OVCAR3 ovarian cancer cells, untreated (considered as a control) or treated with MEL, pure FLV (FLV-R), or FLV-MEL nano-conjugates for 24 h, were measured through the metabolism of MTT [3-(4,5-dimethylthiazol-2-yl)-2,5-diphenyltetrazolium bromide] to a formazan salt as previously described (Caruso et al., 2017; Awan et al., 2020), with slight modifications. Briefly, OVCAR3 cells (1 × 10^5^ cells) were seeded into a 96-well tissue culture plate and incubated in a humidified environment (5% CO_2_, 37°C) in order to ensure the complete attachment of the cells. The day of the experiment, cells were treated for a period of 24 h at the end of which the MTT protocol was applied. The absorbance at 569 nm in each well was read by using a Spark® multimode microplate reader (Tecan Group Ltd., Seestrasse, Maennedorf, Switzerland). The IC50 for each of our experimental conditions (MEL, FLV-R, or FLV-MEL nano-conjugates) was calculated based on the curves obtained measuring the variation of cell viability (%) as a function of increasing concentrations (0.39, 1.56, 6.26, 25 µM, and 100 µM) of MEL, FLV-R, or FLV-MEL nano-conjugates.

### Cell Cycle Analysis

The analysis of cell cycle in OVCAR3 under our experimental conditions was performed by using flow cytometry as previously described (Alfaifi et al., 2020; Fahmy and Aldawsari, 2020). Briefly, OVCAR3 cells, previously seeded in 96-well plates (3 × 10^5^ cells/well), were treated with MEL, FLV-R, or FLV-MEL nano-conjugates for 24 h. Untreated cells were considered as a control. MEL, FLV-R, or FLV-MEL nano-conjugates were used at a sub-toxic concentration (IC10). At the end of the treatment, cells were separated (centrifugation), fixed (70% cold ethanol), centrifuged again, washed (PBS), and stained (propidium iodide + RNase staining buffer). As a last step, each sample was analyzed by using a FACS Calibur flow cytometer (BD Bioscience, United States).

### Annexin V—Propidium Iodide Staining

A dual staining technique was employed to study the impact of our different experimental conditions on the percentage of apoptotic (early or late stages) or necrotic OVCAR3 cells (Hsiao et al., 2016; Alfaifi et al., 2020). OVCAR3 cells, previously seeded in 96-well plates at the density of 3 × 10^5^ cells/well, were treated with MEL, FLV-R, or FLV-MEL nano-conjugates for 24 h. Untreated cells were considered as a control. MEL, FLV-R, or FLV-MEL nano-conjugates were used at a sub-toxic concentration (IC10). Cell staining was accomplished by using Annexin V-FITC Apoptosis Kit (BD Bioscience, CA, United States) using the manufacturer’s suggested protocol, allowing for identifying both early and late phases stages of apoptosis as well as differentiating apoptosis from a cell death that occurred by necrosis. The well-known ability of propidium iodide to penetrate damaged/dead cells only was exploited to distinguish between the two types of cell death.

### BAX and BCL-2 Proteins Determination

The quantitative determination of BAX and BCL-2 proteins was carried out after 24 h treatment by using Human BAX ELISA kit (DRG Instruments GmbH, Marburg, Germany) and Zymed^®^ Bcl-2 ELISA Kit, respectively, according to the manufacturers’ instructions (Fahmy and Aldawsari, 2020; Md et al., 2020). Untreated cells were considered as a control. MEL, FLV-R, or FLV-MEL nano-conjugates were used at a sub-toxic concentration (IC10).

### Mitochondrial Membrane Potential

MitoProbe™ TMRM Assay Kit was used to monitor the changes in MMP occurring in OVCAR3 cells, previously seeded in 96-well plates (1 × 10^5^ cells/well) and exposed to MEL, FLV-R, or FLV-MEL nano-conjugates for 24 h, as previously described (Awan et al., 2020). Untreated cells were considered as a control. MEL, FLV-R, or FLV-MEL nano-conjugates were used at a sub-toxic concentration (IC10).

### Statistical Analysis

The IBM SPSS® statistical (Ver. 25, SPSS Inc., Chicago, IL, United States) or Graphpad Prism (Ver. 8, San Diego, CA, United States) software was used for cell-free and OVCAR3 cells’ experiments, respectively. For multiple comparisons, one-way or two-way ANOVA followed by Tukey’s post hoc test was employed. Each set of experiments is reported as means ± standard deviation (SD) of at least four independent experiments. The statistical significance was set up at *p*-values <0.05.

## Results

### Experimental Design of FLV-MEL

#### Statistical Analysis of the Factorial Design

Establishing the formulation and process variables that could have impact on the drug delivery system features is necessary. Factorial design shows a privilege regarding this aspect as it can analyze the influence of different variables synchronously. In the present study, the variables and their levels were chosen based on preliminary trials. ANOVA was calculated using Type III-partial sum of squares as the design includes only numeric factors. For each response, the predicted *R* ^2^ value fairly agreed with the adjusted *R* ^2^ value for the selected factorial model and process order. Adequate precision was greater than 4 (Table 3), indicating appropriate signal to noise ratio, thus, proving the eligibility of the selected model to navigate the experimental design space (Jaswir et al., 2019; Aldawsari and Badr-Eldin, 2020).

**TABLE 3:** Statistical analysis output of responses data of the 2^3^ factorial design used for formulation of FLV-MEL nanoconjugates.

Responses	Process order	*p*-value	*R* ^2^	Adjusted *R* ^2^	Predicted *R* ^2^	Adequate precision	Significant factors and interactions
Y_1_: particle size (nm)	Main effects	0.0013	0.9740	0.9545	0.8960	14.02	X_3_
Y_2_: zeta potential (mV)	2FI	0.0262	0.9998	0.9986	0.9875	72.91	X_1_, X_2_, X_1_X_2_, X_2_X_3_

Abbreviations: FLV, fluvastatin; MEL, melittin; 2FI, two-factor interaction.

#### Effect of Variables on Particle Size (Y_1_)

The particle size of the prepared FLV-MEL nano-conjugates ranged from 127.25 ± 1.89 to 604.36 ± 4.89 nm (Table 2). According to the factorial design, the factorial model with main effects process order was significant (model F-value = 49.97; *p* = 0.0013). There is only 0.13% chance that an F-value could occur due to noise. The equation representing the main effects in terms of coded factors was generated as follows:

$$Y_{1}=\mathrm{366}\mathrm{.21}+\mathrm{23}\mathrm{.79}\;X_{1}+8\mathrm{.96}\;X_{2}+\mathrm{214}\mathrm{.79}\;X_{3}.$$

**FIGURE 1:**
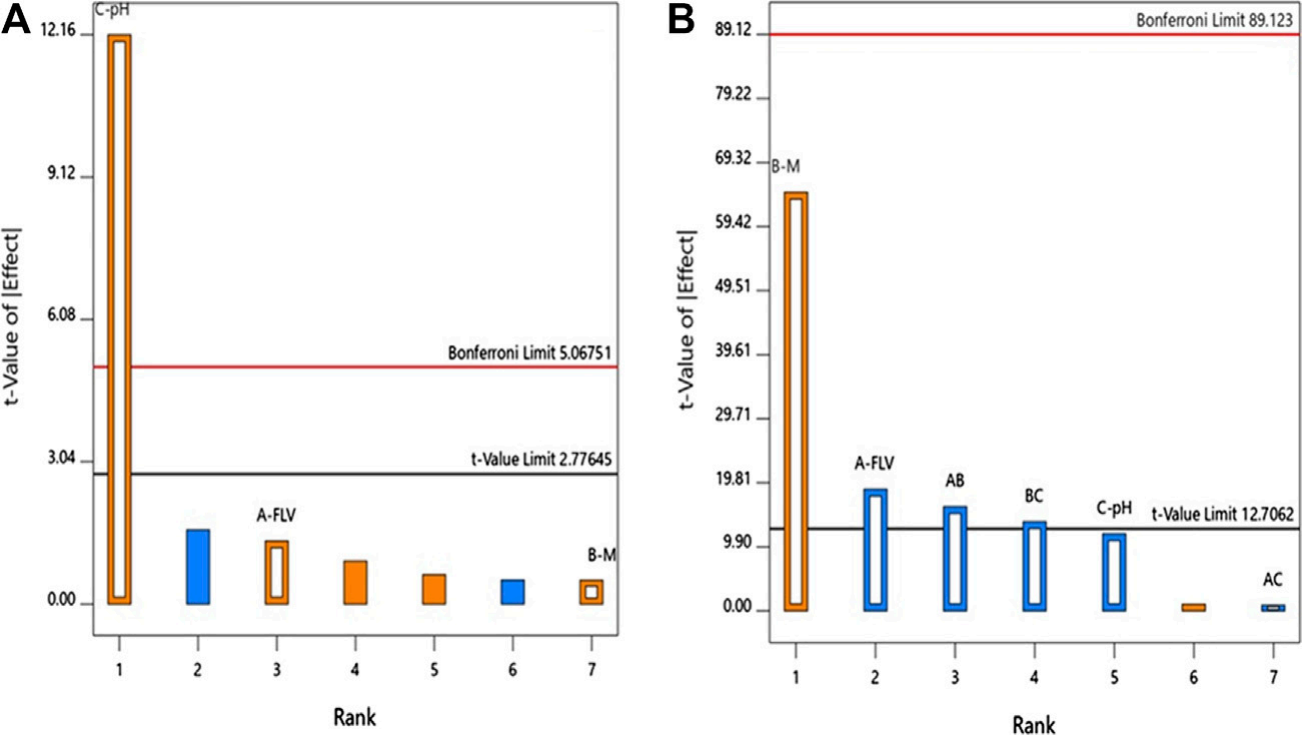
Standardized Pareto Chart for the **(A)** particle size and **(B)** zeta potential of FLV-MEL nano-conjugates.

ANOVA using sum of squares Type III-partial showed that pH (X_3_, *p* = 0.0003) has a positive significant impact on the particle size as evidenced by the positive sign of the term X_3_ coefficient and presented in the Pareto chart, Figure 1A.

**FIGURE 2:**
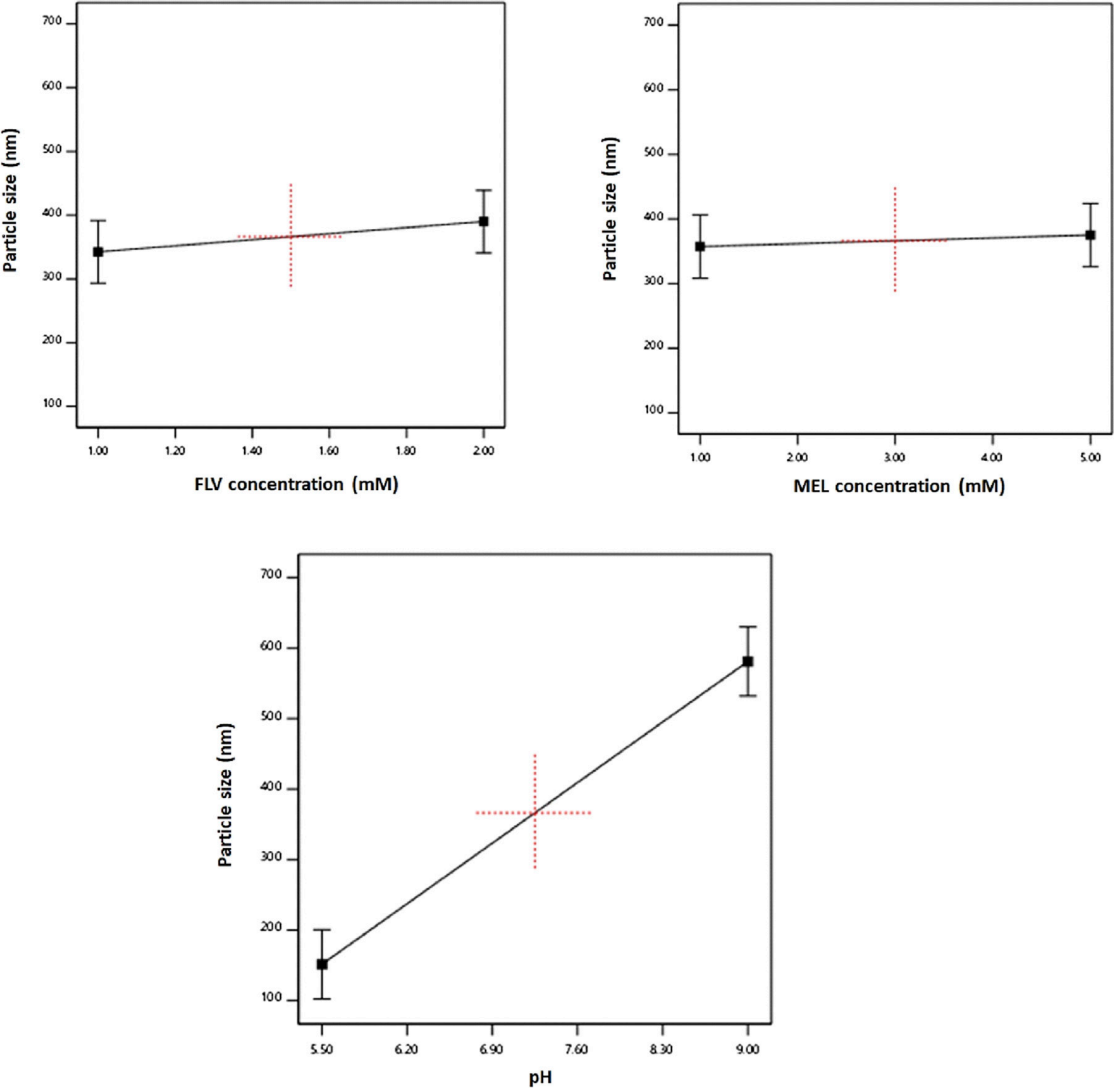
Main effects of FLV (X_1_), MEL concentration (X_2_), and pH (X_3_) on particle size of FLV-MEL nano-conjugates.

The individual effects of the studied factors on the particle size are graphically illustrated in Figure 2. As evident, the size increases with increasing pH value.

#### Effect of Variables on Zeta Potential (Y_2_)

Zeta potential contributes to the charge stabilization of nanoparticulate systems. All the prepared FLV-MEL nano-conjugates showed positive zeta potential ranging from 0.05 ± 0.01 to 17.41 ± 0.42 (Table 2). According to the factorial design, the factorial model with two-factor interaction (2FI) process order was significant (model F-value = 855.79; *p* = 0.0262). There is only 2.62% chance that a F-value could occur due to noise. The equation representing the main effects and interactions in terms of coded factors was generated as follows:

$$Y_{2}=6\mathrm{.12}-1\mathrm{.69}\;X_{1}+5\mathrm{.83}\;X_{2}-1\mathrm{.07}\;X_{3}-1\mathrm{.45}\;X_{1}X_{2}-0\mathrm{.078}\;X_{1}X_{3}-1\mathrm{.24}\;X_{2}X_{3}.$$

ANOVA using sum of squares Type III-partial showed that both FLV (X_2_, *p* = 0.0338) and MEL (X_3_, *p* = 0.0098) concentrations have significant impact on the zeta potential presented in the Pareto chart (Figure 1B). In addition, the interactions terms X_1_X_2_ (*p* = 0.0395) and X_2_X_3_ (*p* = 0.0461) correspond to the interaction between MEL concentration and either FLV concentration or pH, respectively.

**FIGURE 3:**
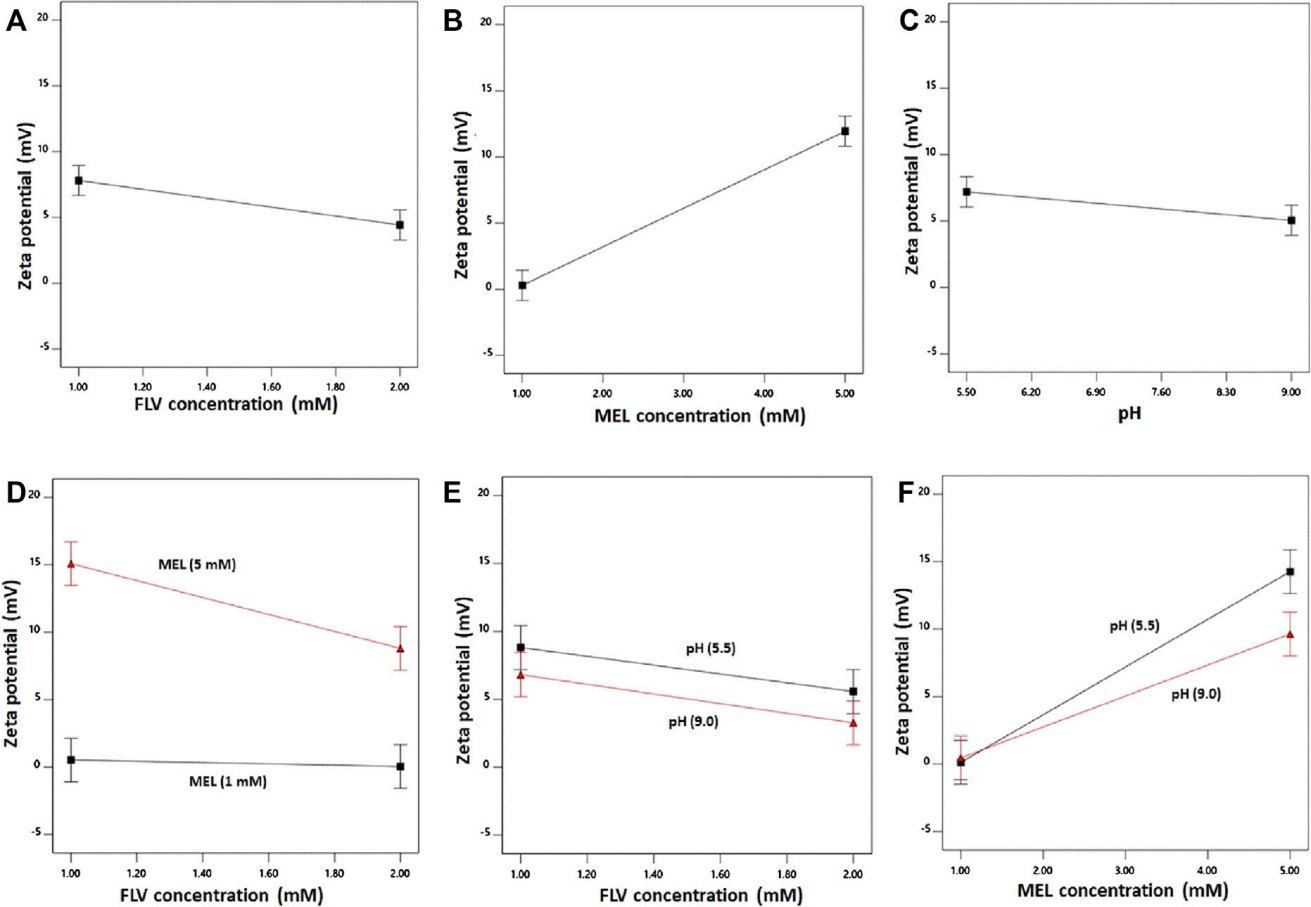
Main effects **(A–C)** and interactions **(D–F)** of concentration FLV (X_1_), MEL concentration (X_2_), and pH (X_3_) on zeta potential of FLV-MEL nano-conjugates.

The individual effects of the studied factors and the 2FI on the zeta potential are graphically illustrated in Figure 3.

As evident, the zeta potential values increase with decreasing drug and increasing MEL concentrations. The effect of MEL was more pronounced on the zeta potential as evidenced by its higher coefficient in the coded equation. Moreover, the effect of the drug was prominent at higher MEL concentration rather than lower one as shown in the interaction graph. The effect of MEL could be attributed to its cationic nature.

### Selection of the Optimized FLV-MEL Nano-Conjugates

Desirability function was utilized for optimal FLV-MEL nano-conjugates selection according to the predetermined goals for the responses. It was found that beads prepared at FLV concentration of 1 mM, MEL concentration of 5 mM, and pH of 5.5 met the required criteria with a desirability value of 0.988. Thus, this formulation was selected for further studies. The proposed formulation was prepared and evaluated for particle size and zeta potential giving results of 136.6 nm and 16.32 mV, respectively. The results were in good agreement with the predicted values with residual percentage of less than 3.32 and 1.89%, respectively.

### Optimized FLV-MEL Nano-Conjugates Display a Strong Cytotoxic Activity in OVCAR3 Ovarian Cancer Cells

**FIGURE 4:**
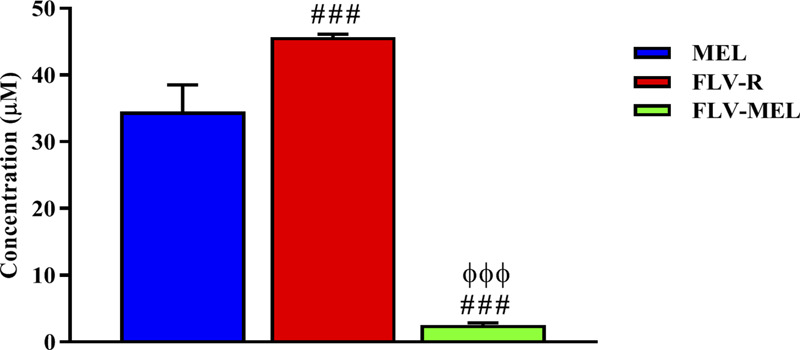
IC50 of the MEL, FLV-R, and FLV-MEL in the OVCAR3 cells. Data are the mean of 4 independent experiments ± SD. The post hoc Tukey test was used for multiple comparisons. ^###^Significantly different vs. MEL (*p* < 0.001). ^ΦΦΦ^Significantly different vs. FLV-R (*p* < 0.001).

Once the optimized formula was obtained, the next aim was to investigate the pharmacological activity and the toxic potential (expressed as IC50) of MEL, FLV-R, or FLV-MEL nano-conjugates treatments (24 h) on OVCAR3 cells. The results obtained by carrying out the MTT assay showed that the highest IC50 value, indicating the lowest toxic potential, belonged to the free drug (FLV-R, IC50 = 45.7 ± 0.4 µM); this IC50 value was even higher than that observed for MEL treatment (IC50 = 34.5 ± 4.0 µM; *p* < 0.001 vs. FLV-R; Figure 4).

The lowest IC50 value, corresponding to the highest toxic potential, was observed in OVCAR3 cells treated for 24 h with FLV-MEL nano-conjugates (IC50 = 2.5 ± 0.3 µM; *p* < 0.001 vs. all; Figure 4).

### FLV-MEL Nano-Conjugates Treatment Inhibits the Proliferation of OVCAR3 Cells

**FIGURE 5:**
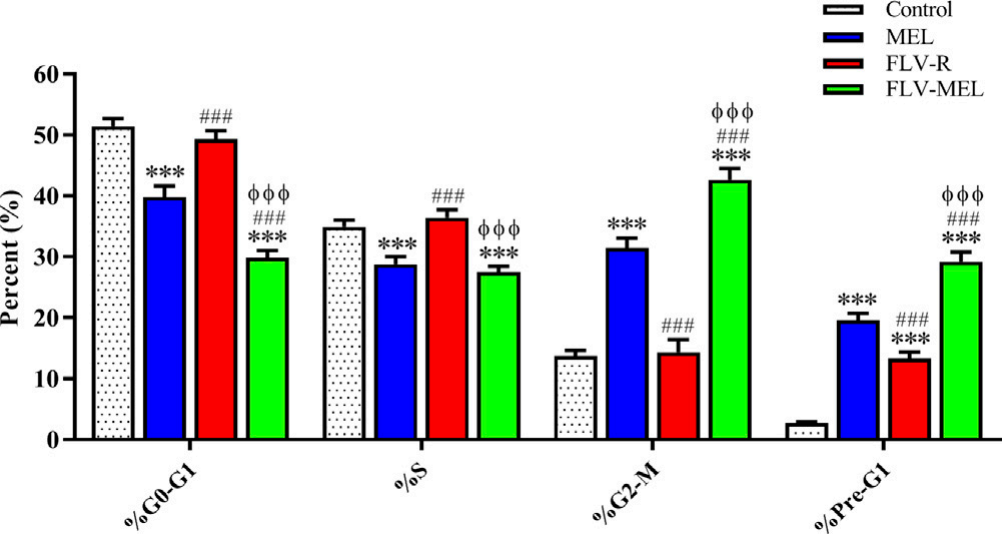
Impact of MEL, FLV-R, or FLV-MEL treatments on OVCAR3 cell cycle phases. Data are the mean of 4 independent experiments ± SD. The post hoc Tukey test was used for multiple comparisons. ***Significantly different vs. control (*p* < 0.001). ^###^Significantly different vs. MEL (*p* < 0.001). ^ΦΦΦ^Significantly different vs. FLV-R (*p* < 0.001).

Figure 5 reports the effects of MEL, FLV-R, or FLV-MEL nano-conjugates treatments (24 h) on OVCAR3 cell cycle phases.

The % values for untreated OVCAR3 cells (control) were 51.4 ± 1.3% for G0/G1, 34.9 ± 1.1% for S, 13.7 ± 0.9% for G2-M, and 2.7 ± 0.1% for pre-G1 phases, clearly depicting quick proliferative properties. FLV-R treatment has not led to any significant changes compared to untreated cells, except in the case of pre-G1 phase (13.3 ± 1.1%; *p* < 0.001 vs. control). Interestingly, MEL, even when not conjugated with the drug, was able to induce significant cell cycle changes regarding all the phases (*p* < 0.001) compared to both untreated and OVCAR3 cells treated with FLV-R. Worth of note are the effects on the cell cycle phases induced by the optimized formula; in fact, the treatment with the optimized FLV-MEL was able to significantly inhibit the proliferation of OVCAR3 cells with a greater extent compared to all the other treatments (*p* < 0.001 vs. all), with % values for G0/G1, S (the only one similar to FLV-MEL), G2-M, and pre-G1 phases equal to 29.8 ± 1.2%, 27.5 ± 0.9%, 42.6 ± 1.9%, and 29.2 ± 1.6%, respectively.

### The Conjugation of FLV to MEL (FLV-MEL) Strongly Enhances the Proapoptotic Potential of the Drug

**FIGURE 6:**
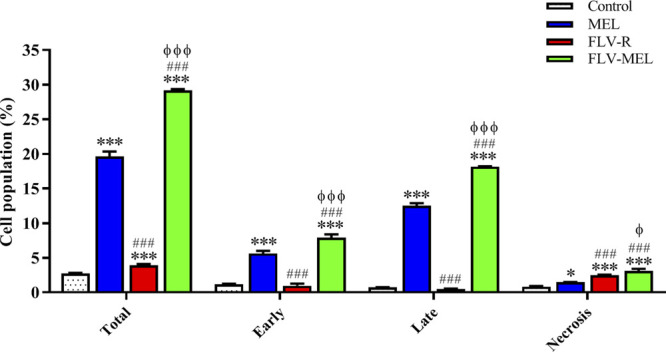
Impact of MEL, FLV-R, or FLV-MEL treatments on the percentage of apoptotic or necrotic OVCAR3 cells. Total = apoptosis + necrosis; Early = early apoptotic phase; Late = late apoptotic phase. Data are the mean of 4 independent experiments ± SD. The post hoc Tukey test was used for multiple comparisons. ***Significantly different vs. control (*p* < 0.001). ^###^Significantly different vs. MEL (*p* < 0.001). ^Φ^Significantly different vs. FLV-R (*p* < 0.05). ^ΦΦΦ^Significantly different vs. FLV-R (*p* < 0.001).

In order to shed more light on the enhanced antiproliferative effect of FLV-MEL and investigate whether it was also accompanied by pro-apoptotic activities, the impact of the different treatments on the percentage of OVCAR3 cells undergoing apoptosis or necrosis was examined. As showed in Figure 6, the treatment with MEL alone was able to significantly increase the percentage of OVCAR3 cells in early (5.6 ± 0.4%; *p* < 0.001) and late (12.5 ± 0.3%; *p* < 0.001) apoptotic stages, as well as of cells undergoing necrosis (1.5 ± 0.1%; *p* < 0.05) compared to untreated (control) cells.

The treatment with the free drug (FLV-R) did not show the same pro-apoptotic ability of MEL against OVCAR3 cells, while it was able to significantly (*p* < 0.001) increase the percentage of cells undergoing necrosis (2.5 ± 0.1%) compared to control cells. As expected based on the results reported in Figure 5, the treatment of OVCAR3 cells with FLV-MEL nano-conjugates significantly increased the percentage of cell population in both early (7.9 ± 0.5%) and late (18.1 ± 0.1%) stages of apoptosis, in necrosis (3.1 ± 0.3%), and in apoptosis + necrosis (total) (29.1 ± 0.2%) compared to all the other experimental conditions (Figure 6), clearly showing the enhanced pro-apoptotic activity of FLV following the combination with MEL in the optimized formula.

### As a Part of Their Pro-Apoptotic Activity FLV-MEL Nano-Conjugates Modulate BAX and BCL-2 Protein Levels

**FIGURE 7:**
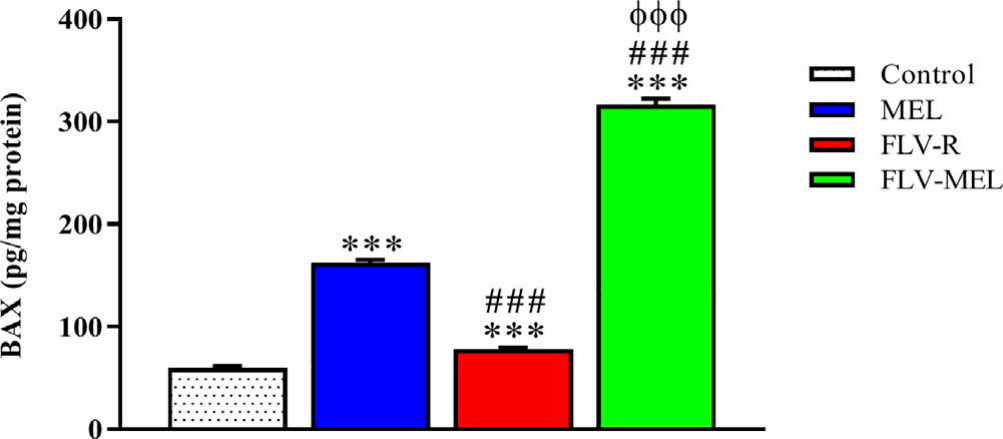
Modulation of MEL, FLV-R, or MEL-FLV treatments on BAX protein concentrations in OVCAR3 cells. Data are the mean of 4 independent experiments ± SD. The post hoc Tukey test was used for multiple comparisons. ***Significantly different vs. control (*p* < 0.001). ^###^Significantly different vs. MEL (*p* < 0.001). ^ΦΦΦ^Significantly different vs. FLV-R (*p* < 0.001).

The expression of BAX protein is related to pro-apoptotic events, while the expression of BCL-2 protein is linked to antiapoptotic activities (Alhakamy and Md, 2019; Faramarzi et al., 2019). Figure 7 depicts the modulation of BAX protein levels in OVCAR3 cells determined by the different experimental conditions.

All the treatments, including FLV-R, were able to significantly (*p* < 0.001) enhance BAX protein levels compared to control cells. MEL treatment significantly enhanced the expression of BAX protein even if compared to FLV-R (*p* < 0.001). The highest effect was observed for OVCAR3 cells treated for 24 h with FLV-MEL nano-conjugates (*p* < 0.001 vs. all the other experimental conditions).

**FIGURE 8:**
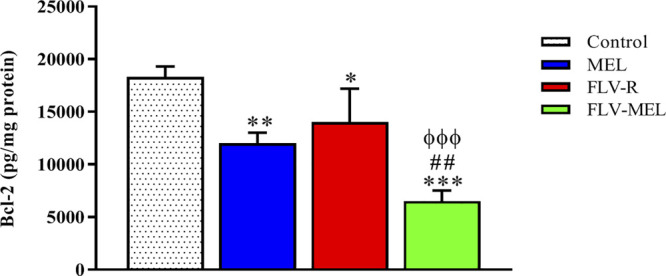
Modulation of MEL, FLV-R, or MEL-FLV treatments on BCL-2 protein concentrations in OVCAR3 cells. Data are the mean of 4 independent experiments ± SD. The post hoc Tukey test was used for multiple comparisons. *Significantly different vs. control (*p* < 0.05). **Significantly different vs. control (*p* < 0.01). ***Significantly different vs. control (*p* < 0.001). ^##^Significantly different vs. MEL (*p* < 0.01). ^ΦΦΦ^Significantly different vs. FLV-R (*p* < 0.001).

A completely opposite pattern compared to the BAX protein trend was observed for BCL-2 protein levels (Figure 8).

As clearly showed in Figure 8, all the treatments have demonstrated the ability to significantly (*p* < 0.001) decrease the levels of BCL-2 protein compared to control cells (*p* < 0.01 for MEL; *p* < 0.05 for FLV-R; *p* < 0.001 for FLV-MEL). Once again, the highest effect, with a synergistic activity of MEL with FLV, was observed when treating OVCAR3 cells with FLV-MEL nano-conjugates (*p* < 0.001 vs. control and FLV-R; *p* < 0.01 vs. MEL).

### The Cytotoxic Activity of FLV-MEL Nanoconjugations Does not Depend on the Modulation of MMP

**FIGURE 9:**
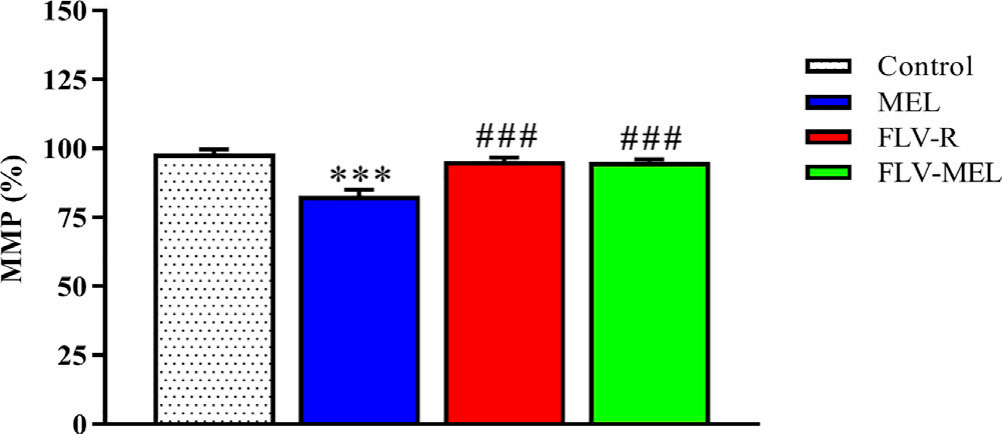
Impact of MEL, FLV-R, or MEL-FLV treatments on the variation of the MMP (%) of OVCAR3 cells. Values were normalized with respect to control untreated OVCAR3 cells and are expressed as the percent (%) variation of MMP. Data are the mean of 4 independent experiments ± SD. The post hoc Tukey test was used for multiple comparisons. ***Significantly different vs. control (*p* < 0.001). ^###^Significantly different vs. MEL (*p* < 0.01).

In order to investigate whether the observed antiproliferative and pro-apoptotic activities of FLV-MEL nanoconjugations were also paralleled by changes in MMP, the variations of the percentages of MMP induced by the different treatments compared to control cells were assessed. Interestingly, neither FLV-R nor FLV-MEL were able to induce detectable alterations in MMP as compared to control, while the treatment with MEL in absence of the drug was able to significantly decrease the MMP (%) (*p* < 0.001 vs. all the other experimental conditions; Figure 9).

## Discussion

FLV is an approved drug, belonging to statins family, able to inhibit HMG-CoA reductase and often prescribed and used for the prevention of adverse cardiovascular events as well as to lower total and LDL cholesterol (Aoki et al., 2012; Adams et al., 2018). This drug (Zhang et al., 2010; Salis et al., 2016; Fahmy, 2018) as well as MEL, the major component of honeybee venom of *Apis mellifera* (Soman et al., 2009), has been shown to exert antineoplastic and toxic effects against different experimental models for cancer, then representing promising approaches for drug discovery processes in cancer. However, adverse effects related to the use of FLV or MEL have been reported, especially in the case when MEL is used at high doses (Saeed and Khalil, 2017; Ramsamooj and Preuss, 2019; Shaw and Kumar, 2019; Ward et al., 2019). The development of an optimized formulation of MEL and FLV in terms of particle size (minimized) and zeta potential (maximized) able to exert a synergistic anticancer activity becomes relevant and then interesting for future translational clinical studies.

The aim of the experimental design and optimization process was to investigate the influence of the variables on the studied responses and to optimize their levels in order to achieve the previously mentioned set goals. Particle size plays a crucial role in the performance of the nanosized particulate delivery systems in the body. Our results revealed that the size increases with increasing pH values. FLV is a weekly acidic antilipemic agent with pK_a_ of approximately 4.5 (Joshi et al., 1999). The increase in the pH value could lead to increased interaction of FLV with MEL that could possibly increase the size of the conjugate. The zeta potential values increase with decreasing drug and increasing MEL concentrations. The effect of MEL could be attributed to its cationic nature. As previously mentioned, MEL is a 26-amino acid cationic bee venom peptide that has six positive charges and no negative charges. Most of the positively charged residues occur at the C-terminal segment of the molecule yielding a net charge of +6 (Ramalingam et al., 1992; Pincus, 2001; Therrien et al., 2016). On the other hand, as previously indicated, FLV is a weekly acidic agent; thus, as the concentration of the drug increases, the interaction between the negative charge of the drug and the cationic C-terminal segments of the MEL could increase leading to decreased overall surface charge and zeta potential of the formed nano-conjugates.

Once the optimized formula was obtained, we carried out *in vitro* experiments in which the ability of MEL to enhance the antineoplastic activity of a sub-toxic concentration of FLV against OVCAR3 cells, representing a well-known experimental model to study the toxic potential of statins alone or in combination with other antineoplastic drugs against ovarian cancer (Scoles et al., 2010; Casella et al., 2014; Abdullah et al., 2019; Yarmolinsky et al., 2020), was tested. The first indication of an improved therapeutic potential of FLV-MEL nano-conjugates comes from the data showed in Figure 4, regarding the different values of the IC50, often used to compare the antiproliferative activity and the toxic potential of different anticancer agents (Schaffrath et al., 2017), under our experimental conditions. The IC50 of FLV-MEL nano-conjugates (2.5 ± 0.3 µM) was significantly lower than that observed for FLV alone (FLV-R) (45.7 ± 0.4 µM) or MEL (34.5 ± 4.0 µM). This synergistic cytotoxic effect obtained by the conjugation of FLV to MEL (FLV-MEL nano-conjugates) is of great relevance from different point of views. In fact, despite the widespread use of statins for conventional (Zhou and Liao, 2009; Islam et al., 2017; Ward et al., 2019) and non-conventional [e.g., cancer treatment (Stancu and Sima, 2001; Koyuturk et al., 2007; Kwan et al., 2008; Ahern et al., 2011; Manthravadi et al., 2016; Van Wyhe et al., 2017)] therapies, clinically relevant adverse effects have been observed even at therapeutic doses (Mach et al., 2018; Newman et al., 2019; Ward et al., 2019); on the other hand, notwithstanding its anticancer activity, the use of MEL in clinical practice raises many concerns as a consequence of its non-specific cytotoxicity and hemolytic activity when used at high doses (Shaw and Kumar, 2019). Hence, the development of new formulations where the synergistic antineoplastic activity of FLV and MEL can be combined in an optimized formula offers the potential for enhancing therapeutic effects, simultaneously reducing the incidence of adverse drug events.

Additional proofs of the enhanced anticancer activity of FLV-MEL nano-conjugates compared to all the other experimental conditions were observed when examining the effects of the different treatments on OVCAR3 cell cycle phases (Figure 5). As clearly depicted, FLV-MEL nano-conjugates treatment strongly inhibited the proliferation of OVCAR3 cells, measured by the reduction of G1→S transition as well as the inhibition of the transition from G2 to M phase. These effects could depend on the ability of MEL to suppress the proliferation of cancer cells (Tipgomut et al., 2018; Ceremuga et al., 2020; Yao et al., 2020) which acts synergistically with FLV (Hillyard et al., 2002; Garwood et al., 2010; Hwang et al., 2017) even when used at a sub-toxic concentration.

The pro-apoptotic and pronecrotic effects of the conjugation of FLV to MEL were then investigated. As expected based on the results regarding the changes in cytotoxicity and antiproliferative activity of OVCAR3 cells under our experimental conditions, FLV-MEL nano-conjugates significantly increased the percentage of cell population in both early and late stages of apoptosis, in necrosis, and in the total cell death (apoptosis + necrosis) compared to FLV-R or MEL (Figure 6). These results are in agreement with previous studies showing the ability of FLV to induce apoptosis also in different non-neoplastic cell types such as vascular endothelial cells (Newton et al., 2003), immune T cells (Samson et al., 2005), and mast cells (Paez et al., 2020). It is also worthwhile to point out that FLV pro-apoptotic activity is exerted at a very low (sub-toxic) concentration and is due to its synergistic combination with MEL, as demonstrated by the very low activity of the free drug (FLV-R). The results on pro-apoptotic activity of FLV-MEL were also strengthened by the ability of this synergistic formula to modulate BAX (increase) (Figure 7) and BCL-2 (decrease) (Figure 8) protein levels in OVCAR3 cells. This increased BAX/BCL-2 ratio is in line with previous finding by Qi et al. showing the ability of FLV to induce cell death of lymphoma cells through the enhanced activation of pro-apoptotic members such as caspase-3 and BAX, simultaneously suppressing BCL-2 (Qi et al., 2013).

Interestingly, neither FLV-R nor FLV-MEL showed the ability to induce any detectable changes in MMP of OVCAR3 cells compared to untreated cells; in fact MEL treatment only was able to significantly decrease the MMP (%) (Figure 9). This could depend on the MEL peptide structure and its cationic nature (six positive charges). Among the different mechanisms regulating the proliferative status of OVCAR3 cells, FLV-MEL could exert anticancer effects especially by promoting pro-apoptotic/necrotic phenomena rather than modulating the MMP.

Despite the significantly highest antineoplastic activity showed by FLV-MEL combination, the activity of MEL alone is also worth of mention. In fact, as showed in almost all *in vitro* cell experiments, the antineoplastic activity of MEL alone used at a sub-toxic concentration (IC10) was higher than that of the free drug (FLV-R) and is in accordance with several recent research studies (Sangboonruang et al., 2020; Yu et al., 2020) such as that carried out by Duffy et al. showing the ability of MEL to suppress epidermal growth factor receptors 1 and 2 (EGFR and HER2) activation in the aggressive triple-negative and HER2-enriched breast cancer subtypes (Duffy et al., 2020). The present findings also confirm that the combination of this natural product with other antineoplastic drugs results in a more powerful anticancer activity.

Conventional drug delivery system cannot deliver the antineoplastic agents in the most effective concentration to cause tumor cell death, and debilitating side effects occur. The combination of two antineoplastic agents in nano-conjugates represents a novel path to improve the therapeutic index and pharmacokinetic profile of chemotherapeutic agents (Fernandes et al., 2018). After considering the physicochemical properties of our FLV-MEL nano-conjugates (size, surface properties, and stability), we believe that these nano-conjugates, when administered intraperitoneally, can comply in a highly efficient way with the five steps of the CAPIR cascade: blood Circulation, Accumulation and Penetration in the tumor, cell Internalization, and intracellular Release of the drug (Tay et al., 2014). Future *in vivo* studies are required for a better understanding of pharmacokinetic profile of FLV-MEL nano-conjugates in animal models of ovarian cancer.

## Conclusion

In the present study, a two-level, three-factor (2^3^) full factorial design was employed for the preparation of FLV-MEL nano-conjugates as well as for their optimization, obtained minimizing the particle size and maximizing the magnitude of the zeta potential. The *in vitro* experiments carried out on OVCAR3 ovarian cancer cells clearly demonstrated the synergistic anticancer activity of FLV-MEL nano-conjugates characterized by an enhanced toxic (lower IC50) and antiproliferative potential. This formulation containing a sub-toxic concentration of FLV also pro-apoptotic pronecrotic and proapoptotic activities, the latter mediated by the modulation of BAX/BCL-2 ratio. Our optimized FLV-MEL formulation showing a synergistic anticancer activity might therefore represent a novel path for the development of specific and more effective antineoplastic drugs directed against ovarian cancer.

## Data Availability Statement

The raw data supporting the conclusion of this article will be made available by the authors, without undue reservation.

## Author Contributions

SB-E, FC, and GC gave substantial contributions to the conception and design of the work. AAA, HMA, WR, WM, AFA, and SA supervised the experiments regarding the preparation and optimization of FLV-MEL nano-conjugates. SB-E, NA, UF, and HZA performed the experiments. SB-E, NA, UF, and GC analyzed the data. OA, HZA, AAA, HMA, WR, WM, AFA, and SA participated in the design of the study. SB-E, NA, UF, AAA, HMA, WR, WM, AFA, SA, and GC carried out the statistical analysis and prepared the tables and/or figures. SB-E, FC, and GC drafted the work. The first draft of the manuscript was reviewed/edited by all the authors. All authors listed have read and agreed to the final version of the manuscript.

## Funding

This project was funded by the Deanship of Scientific Research (DSR) at King Abdulaziz University, Jeddah, under grant no. RG-3-166-41.

## Conflict of Interest

The authors declare that the research was conducted in the absence of any commercial or financial relationships that could be construed as a potential conflict of interest.
